# Experimental Research on Shipborne SINS Rapid Mooring Alignment with Variance-Constraint Kalman Filter and GNSS Position Updates

**DOI:** 10.3390/s24113487

**Published:** 2024-05-28

**Authors:** Zhipeng Fan, Hua Chai, Xinghui Liang, Hubiao Wang

**Affiliations:** 1 State Key Laboratory of Geodesy and Earth’s Dynamics, Innovation Academy for Precision Measurement Science and Technology, Chinese Academy of Sciences, Wuhan 430000, China; fanzp@apm.ac.cn (Z.F.); lxh_whigg@whigg.ac.cn (X.L.); wanghb@whigg.ac.cn (H.W.); 2 College of Earth and Planetary Sciences, University of Chinese Academy of Sciences, Beijing 100000, China

**Keywords:** inertial navigation system (INS), fine alignment, Kalman filter, variance constraint, shipborne inertial systems

## Abstract

Analytical coarse alignment and Kalman filter fine alignment based on zero-velocity are typically used to obtain initial attitude for inertial navigation systems (SINS) on a static base. However, in the shipboard mooring state, the static observation condition is corrupted. This paper presents a rapid alignment method for SINS on swaying bases. The proposed method begins with a coarse alignment technique in the inertial frame to obtain an initial rough attitude. Subsequently, a Kalman filter with position updates is employed to estimate the remaining misalignment error. To enhance the filter estimation performance, an appropriate lower boundary is set to the target states’ variances according to a carefully designed relative convergence index. The variance-constraint Kalman filter (VCKF) approach is proposed in this paper, and the shipborne experiments validate its effectiveness. The results demonstrate that the VCKF approach significantly reduces the time requirement for fine alignment to achieve the same accuracy on a swaying base, from 90 min in the classic Kalman filter to 30 min. Additionally, the parameter estimation performance in the Kalman filter is also improved, particularly in situations where unpredicted external interference is involved during fine alignment.

## 1. Introduction

Currently, the initial attitude for a navigation-grade strapdown inertial navigation system (SINS) on a static base is obtained through two sequential steps: analytical coarse alignment and zero-velocity Kalman filter fine alignment [1,2]. However, in shipborne mooring conditions, the static observation condition can be easily corrupted by sea swell and surge, which affects the readings of the gyroscopes and accelerometers, leading to the failure of analytical coarse alignment validated on a static base [3]. Moreover, the zero-velocity condition required by the Kalman filter for fine alignment cannot be strictly satisfied in this case.

Two main types of solutions are currently employed to address the problem of analytical coarse alignment failure. The first type focuses on extending the applicable scope of coarse alignment methods, such as performing coarse alignment in the inertial frame. This method utilizes gyroscope readings to track angular motion, reducing the influence of swaying on subsequent gravity vector integration. As a result, a rough attitude that satisfies the assumption of a small misalignment angle is obtained for further Kalman Filter fine alignment [3,4,5,6,7,8,9]. The second type aims to increase the tolerance of the initial attitude error in the Kalman Filter by establishing a large misalignment angle error model. Nonlinear filter methods, such as the extended Kalman filter (EKF), the unscented Kalman filter (UKF), or the cubature Kalman filter (CKF), are employed for fine alignment [10,11,12,13,14,15,16,17,18]. Although these methods do not require a small initial misalignment angle assumption, the mathematical models involved are relatively complex, and convergence may take longer in the presence of a large initial azimuth error [19].

Regarding the inability to strictly achieve the zero-velocity condition in the mooring state, some scholars suggest using external equipment, like the Doppler Velocimetry Log (DVL), to provide velocity observations for the Kalman filter. However, when Global Navigation Satellite System (GNSS) signals are available, position observations can also be used as external updates for misalignment estimation [4]. Compared to velocity updates, position updates offer certain advantages for alignment applications in the Kalman filter. Firstly, position observations represent the integral of velocity over time, and the integration process acts as a low-pass filter, providing stronger resistance to high-frequency interference. Consequently, more accurate alignment results are expected [1]. Secondly, the change in position during fine alignment, known as line motion, directly corresponds to external observations from GNSS, potentially reducing the impact of line motion when using position updates [3].

In this study, GNSS position updates are utilized to achieve SINS fine alignment in the shipborne mooring environment. Initially, the small misalignment condition is obtained through inertial frame coarse alignment, followed by estimation of attitude error using the Kalman filter. The closed-loop feedback is then employed to compensate for the remaining misalignment. However, poor observability in azimuth misalignment poses a constraint on alignment speed [20], potentially prolonging the fine alignment time. Additionally, any burst interference during the alignment also affects the accuracy of misalignment estimation. With the expectation of reducing the time of misalignment estimation and improving the stability of the Kalman filter, the variance-constraint Kalman filtering (VCKF) method is proposed in this study. This method introduces a relative convergence index and sets up variance constraint criteria based on observability analysis. This paper describes the VCKF method and demonstrates its application on SINS fine alignment in a shipborne mooring base.

The organization of this paper is structured as follows: In Section 2, the inertial frame coarse alignment method is described. Following that, a Kalman filter based on position observation and a closed-loop feedback strategy is adopted for fine alignment in Section 3. The concept of relative convergence degree and VCKF method that aims to enhance the filter estimation performance is introduced in Section 4, and the corresponding experiment results are exhibited in Section 5. In Section 6, a conclusion is drawn for this paper.

## 2. Inertial Frame Coarse Alignment for Mooring State

The primary objective of coarse alignment is to obtain an initial approximate attitude in the mooring state for subsequent fine alignment. In this study, a technique called inertial frame coarse alignment was employed, in which the gyro is used to track the angular motion of the vessel, and two velocity or position vectors are constructed separately by integrating the gravity vector over a certain duration to determine the initial attitude.

Given that the position vector calculated from quadratic integration exhibits stronger resistance to movement interference in the mooring state, we chose the position-type vector for this study. The principle can be expressed using Equation (1) [6], which splits the transformation matrix into the multiplication of several transformation matrices.

(1) $$C_{b}^{n}=C_{e}^{n}C_{i}^{e}C_{i_{b0}}^{i}C_{b}^{i_{b0}}$$

where $C_{b}^{n}$ is direction cosine matrix from b-frame to n-frame; the superscript n refers to the ENU frame; subscripts b,e, and i denote body frame, ECEF frame, and inertial frame, respectively; and $i_{b0}$ is the inertial frame that fix to the body frame of time 0.

Here, the transformation matrices $C_{e}^{n}$ and $C_{i}^{e}$ are obtained as follows:

(2) $$C_{e}^{n}=\left[\begin{array}{ccc} 0 & 1 & 0\\ -\sin L & 0 & \cos L\\ \cos L & 0 & \sin L \end{array}\right]$$

(3) $$C_{i}^{e}=\left[\begin{array}{ccc} \cos\omega_{ie}\left(t-t_{0}\right) & \sin\omega_{ie}\left(t-t_{0}\right) & 0\\ -\sin\omega_{ie}\left(t-t_{0}\right) & \cos\omega_{ie}\left(t-t_{0}\right) & 0\\ 0 & 0 & 1 \end{array}\right]$$

In which L is the latitude, and $\omega_{ie}$ is the angular rate of the Earth’s rotation.

At the time $t_{0}$, $C_{b}^{i_{b0}}$ is a unit matrix later updated with gyroscope output [6]. Since the gyros can effectively track angular motion, the inertial frame coarse alignment remains valid even in the presence of vessel angular motion.

The challenge now is to achieve the $C_{b}^{n}$ matrix in Equation (1) by solving for the $C_{i_{b0}}^{i}$. The solution of the $C_{i_{b0}}^{i}$ matrix is given by [20],

(4) $$C_{i_{b0}}^{i}={\left[\begin{array}{c} ({P_{t_{1}}^{i})}^{T}\\ {\left(P_{t_{1}}^{i}\times P_{t_{2}}^{i}\right)}^{T}\\ {\left(P_{t_{1}}^{i}\times P_{t_{2}}^{i}\times P_{t_{1}}^{i}\right)}^{T} \end{array}\right]}^{-1}\left[\begin{array}{c} {\left(P_{t_{1}}^{i_{b0}}\right)}^{T}\\ {\left(P_{t_{1}}^{i_{b0}}\times P_{t_{2}}^{i_{b0}}\right)}^{T}\\ {\left({P_{t_{1}}^{i_{b0}}\times \omega }_{\mathrm{ie}}^{b}\times P_{t_{1}}^{i_{b0}}\right)}^{T} \end{array}\right]$$

where

(5) $$P_{t_{1}}^{i}=\iint_{t_{0}}^{t_{1}}g^{i}dt$$

$g^{i}$ is gravitational acceleration in inertial frame.

(6) $$P_{t_{1}}^{i_{b0}}=\iint_{t_{0}}^{t_{1}}({-C_{b}^{i_{b0}}f}^{b}+{C_{b}^{i_{b0}}(a}^{b}+\nabla^{b}))dt$$

In Equation (6), $f^{b}$ is the accelerometer output, $a^{b}$ represents the interference acceleration caused by movement, and $\nabla^{b}$ is the bias from accelerometers. These factors are usually neglected in the coarse alignment phase.

Referring to Equations (5) and (6), the solutions of $P_{t_{2}}^{i}$ and $P_{t_{2}}^{i_{b0}}$ in Equation (4) can be obtained in the same way.

## 3. Kalman Filter Misalignment Estimation Based on Position Update

Once we acquired the rough initial attitude from coarse alignment, the next step was to conduct fine alignment using the Kalman filter method to precisely determine the attitude transformation matrix before sailing.

A 15-state Kalman filter equation, similar to SINS/GNSS combination navigation, was adopted for fine alignment with position update. The state vectors include attitude misalignments (ϕ), velocity errors (δv), position errors (δL,δλ,δh), gyroscopic drifts (ε), and accelerometer biases (∇).

$$X=[\phi_{E},\phi_{N},\phi_{U},\delta v_{E},\delta v_{N},\delta v_{U},\delta L,\delta \lambda ,\delta h,\varepsilon_{E},\varepsilon_{N},\varepsilon_{U},\nabla_{E},\nabla_{N},\nabla_{U}]$$

The error equation of the Kalman filter includes the attitude error equation,

(7) $$\dot{\phi }=\Omega \times \omega_{in}^{n}+\delta \omega_{in}^{n}-\delta \omega_{ib}^{n}$$

In which Ω represents the skew–symmetric matrix of ϕ.

The velocity error equation is as follows:

(8) $$\begin{array}{cc} \delta \dot{V}= & \Omega \times f^{b}+{\hat{C}}_{b}^{n}\delta f^{b}-(2\delta \omega_{ie}^{n}+\delta \omega_{en}^{n})\times V^{n}\\ & -(2\omega_{ie}^{n}+\omega_{en}^{n})\times \delta V^{n}+\delta g^{n} \end{array}$$

The position error equation is as follows:

(9) $$\begin{array}{c} \delta \dot{L}=\frac{1}{R_{M}+h}\delta v_{N}-\frac{v_{N}}{{\left(R_{M}+h\right)}^{2}}\delta h\\ \delta \dot{\lambda }=\frac{\sec L}{R_{N}+h}\delta v_{E}+\frac{v_{E}\sec L\tan L}{R_{N}+h}\delta L-\frac{v_{E}\sec L}{{\left(R_{N}+h\right)}^{2}}\delta h\\ \delta \dot{h}=\delta v_{U} \end{array}$$

Gyro drifts and accelerometer biases are modeled as constants.

(10) $$\dot{\varepsilon }=0$$

(11) $$\dot{\nabla }=0$$

Directly writing the Equations (7) to (11) into matrix form and then discretizing yields

(12) $$X_{k/k-1}=\Phi_{k/k-1}X_{k-1}+\Gamma_{k-1}W_{k-1}$$

Here, $\Phi_{k/k-1}$ and $\Gamma_{k-1}$ are the state transition matrix and the system noise distribution matrix, respectively, which are consistent with the literature [19]; $W_{k-1}$ is the system noise vector.

The observation equation of the Kalman filter with position updates is given by:

(13) $$Z_{k}={[p_{INS}-p_{GNSS}]=H}_{k}X_{k}+V_{k}$$

(14) $$H_{k}=\left[0_{3\times 6},I_{3\times 3},0_{3\times 6}\right]$$

where $p_{INS}$ and $p_{GNSS}$ represent SINS and GNSS positions, respectively, including longitude, latitude, and altitude. $H_{k}$ is the observation matrix, and $V_{k}$ is the observation noise vector.

**Figure 1:**
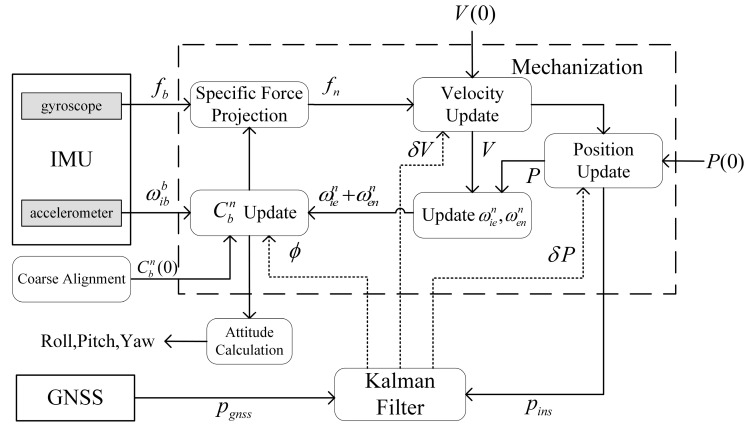
Closed-loop feedback for Kalman filter misalignment estimation with position update.

Compensating for the estimated error from the Kalman filter is crucial for fine alignment. In this study, as shown in Figure 1, a closed-loop feedback strategy is adopted, where the position, velocity, and misalignment errors are simultaneously fed back to account for the linear assumption of the filtering system being disrupted when errors accumulate excessively.

## 4. Variance-Constraint Kalman Filter Based on Relative Convergence Degree

The variance of estimated states in a Kalman filter typically decreases over time in principle [21]. However, according to observability theory [22,23,24], the equivalent eastward gyro drift and two horizontal accelerometer biases are usually considered unobservable in a 15-state Kalman filter that is used in this study for fine alignment. It is nearly impossible for these unobservable states to converge to the correct values during the filtering process. On the other hand, the remaining error states are considered observable even though their convergence rates and variances may vary.

To characterize the convergence of each state’s variance over time, we utilize the ratio of each state’s standard deviation to its initial value, calculated as follows in a dimensionless manner:

(15) $$\sigma_{k\left(j\right)}=\sqrt{\frac{P_{k\left(jj\right)}}{P_{0\left(jj\right)}}}$$

**Figure 2:**
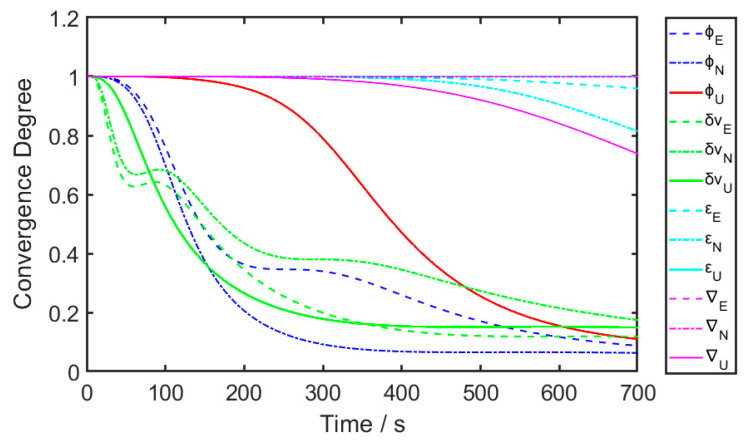
The convergence of filtering states’ variances.

The convergence of each state’s variance from a shipborne test is demonstrated in Figure 2.

Figure 2 aligns with the conclusions drawn from the observability analysis. It illustrates that the velocity error and horizontal misalignment exhibit the fastest convergence rates, indicating their strong observability. In contrast, the azimuth misalignment error converges notably slower, reflecting its lower observability. Moreover, the convergence of gyroscope drift and accelerometer bias is even slower than that of the azimuth misalignment.

As the state variance initially converges, the Kalman filter gradually tends to trust the predictions, thereby reducing the weight of external observations in the filtering result accordingly. This may have two effects. Firstly, considering that some observable states, such as azimuth misalignment, have not yet been fully estimated at this point, the decrease in the weight of external observations affects the estimation efficiency of these parameters. Secondly, when there are obvious environmental disturbances during the alignment process, the error model based on linear assumptions may fail to describe the actual motion accurately. The over trust in the predictions, rather than external observations, in this case, may probably lead to inappropriate filtering results.

**Figure 3:**
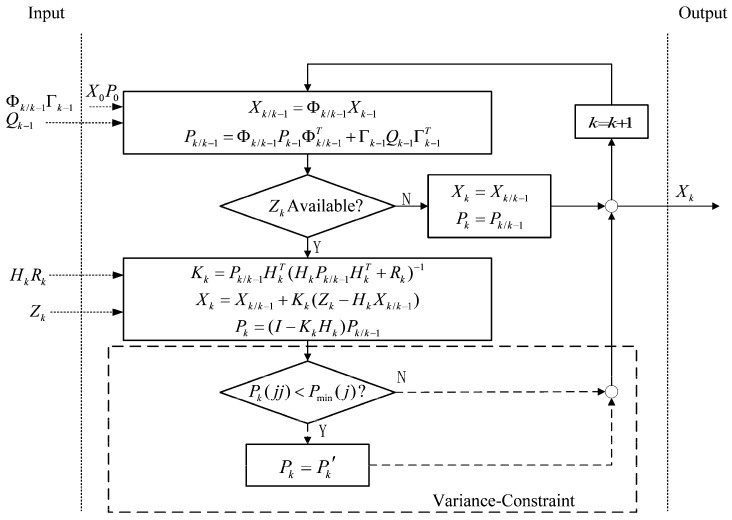
Process of variance-constraint Kalman filter.

To prevent such incidents and expedite the estimation of azimuthal error, it is recommended to incorporate lower bound constraints on the variance of error states associated with poor observability, which include azimuth misalignment, gyro drift, and accelerometer bias. This approach involves adding extra noise to specific target states, thereby creating an “incentive effect” to ensure the effective utilization of external observations following the initial convergence, and it is the so-called VCKF method proposed in this paper for shipborne SINS rapid fine alignment, with its implementation elaborated in Figure 3.

As illustrated, the primary difference between the VCKF and the classic KF is that once the main diagonal element $P_{k\left(jj\right)}$ is smaller than its lower limit $P_{min(j)}$, then it is replaced by its lower limit $P_{min(j)}$.

(16) $$P_{k\left(jj\right)}=max\{P_{k\left(jj\right)},P_{min}\left(j\right)\}$$

A new variance matrix $P_{k}^{\prime }$ is constructed to participate in subsequent filtering.

(17) $$P_{k}^{\prime }=\left[\begin{array}{cc} \begin{array}{ccc} P_{k(11)} & \cdots & P_{k(1j)}\\ \vdots & \ddots & \vdots \\ P_{k(j1)} & \cdots & P_{min(j)} \end{array} & \begin{array}{cc} \cdots & P_{k(1n)}\\ \ddots & \vdots \\ \cdots & P_{k(jn)} \end{array}\\ \begin{array}{ccc} \vdots & \ddots & \vdots \\ P_{k(n1)} & \cdots & P_{k(nj)} \end{array} & \begin{array}{cc} \ddots & \vdots \\ \cdots & P_{k(nn)} \end{array} \end{array}\right]$$

Equation (16) can be further decomposed into

(18) $$P_{k}^{\prime }=\left[\begin{array}{ccccc} P_{k(11)} & \cdots & P_{k(1j)} & \cdots & P_{k(1n)}\\ \vdots & \ddots & \vdots & \ddots & \vdots \\ P_{k(j1)} & \cdots & P_{k(jj)} & \cdots & P_{k(jn)}\\ \vdots & \ddots & \vdots & \ddots & \vdots \\ P_{k(n1)} & \cdots & P_{k(ji)} & \cdots & P_{k(nn)} \end{array}\right]+\left[\begin{array}{ccccc} 0 & \cdots & 0 & \cdots & 0\\ \vdots & \ddots & \vdots & \ddots & \vdots \\ 0 & \cdots & P_{min(j)}-P_{k(jj)} & \cdots & 0\\ \vdots & \ddots & \vdots & \ddots & \vdots \\ 0 & \cdots & 0 & \cdots & 0 \end{array}\right]$$

It is not difficult to be aware that the incentive effect is implemented by imposing noise $P_{\min\left(j\right)}-P_{k\left(jj\right)}$ to target diagonal elements.

Determining the lower boundary value $P_{min}$ is a crucial step in the VCKF method. We propose finding the lower boundary value on the original convergence curve of the Kalman filter, using the relative convergence degree of variance as the criterion defined in Equation (19).

(19) $$\sigma_{k\left(ij\right)}=\frac{\sigma_{k\left(j\right)}}{\sigma_{k\left(i\right)}}$$

**Figure 4:**
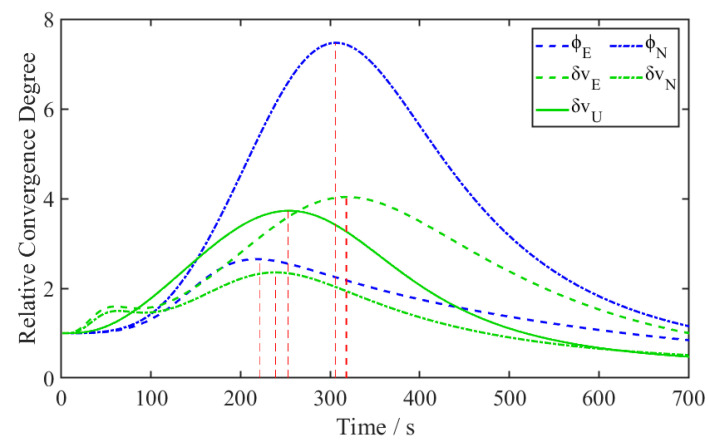
The relative convergence degree of the other five states with respect to the azimuth misalignment.

The fine alignment aims at converging the azimuth misalignment, and therefore, it is necessary to focus on the convergence degree of each state relative to the azimuth misalignment $\sigma_{k\left(i3\right)}$. Using the same data as in Figure 2, we plotted the relative convergence degree of each strong observable state to the azimuth misalignment in Figure 4, in which the peak of $\delta v_{E}$ appears the latest. According to observability theory, strong observable states should have converged at this time point, whereas the azimuth misalignment has not yet fully converged, not to mention those remaining states with even lower observability or that are unobservable. We selected the states that have not yet achieved full convergence and the unobservable ones as target states for applying a lower-bound constraint. Once the final peak of strong observable states is identified, the variances in the target states are restrained to the values at this specific moment for subsequent Kalman filter processing.

It is important to note that the calculation of relative convergence relies solely on the variance of each state. Except for the unstable stage at the beginning, once the relative convergence profile continuously declines in time, the moment at which the maximum relative convergence occurs can be determined. Therefore, this method can be implemented in near real-time in practical applications.

## 5. Shipborne Mooring Alignment Experiments

**Figure 5:**
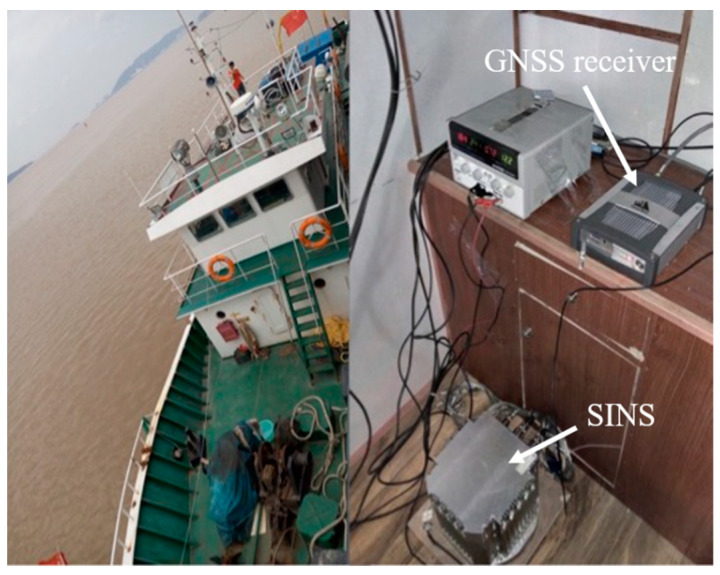
Experiment environment and devices.

In this section, we conduct tests using actual shipborne data and in a dynamic sea environment. Three groups of tests were performed to verify the performance of our proposed method in initial alignment under different sea and swaying conditions. The experiment environment and devices are illustrated in Figure 5. We utilized a high-precision ring laser gyros SINS with gyro drift better than 0.005°/h and accelerometer stability better than 10 μg. The SINS sampling rate was set at 200 Hz.

Since it is challenging to assess the accuracy of initial alignment directly in a mooring and swaying state, we indirectly evaluated the initial alignment accuracy by analyzing pure inertial navigation error after alignment was completed.

### 5.1. Test for Inertial Frame Coarse Alignment in Moored State

The tests in this section evaluate the performance of the inertial frame coarse alignment method in a mooring environment. The total duration of SINS observations is approximately 4.5 h, with the initial 0.5 h dedicated to initial alignment and the subsequent 4 h for navigation. The vessel remained moored throughout the test, experiencing only slight swaying without significant displacement.

**Figure 6:**
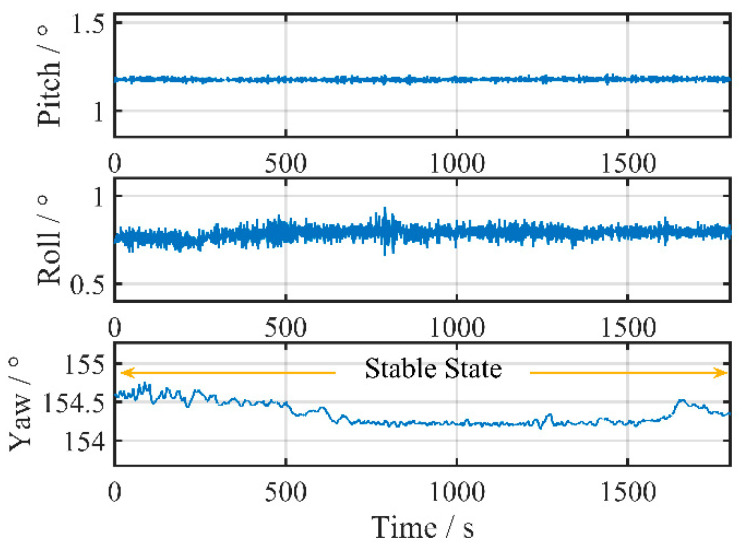
Attitude changes during the coarse alignment test.

The angular motion caused by disturbances in the ocean and other external factors was observed by the gyroscope and reflected in the change in attitude. Therefore, the alignment environment during initial alignment can be indirectly described by the change in vessel attitude. Figure 6 illustrates the changes in vessel attitude, which appear relatively stable without prominent periodic disturbances during this set of tests.

**Figure 7:**
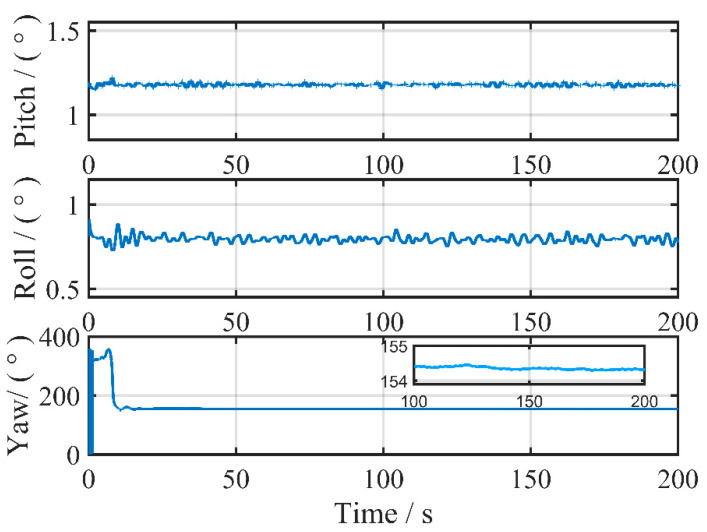
Inertial frame coarse alignment attitude convergence in 200 s.

We employed the inertial frame coarse alignment scheme described in Section 2 to obtain the rough initial attitude of the vessel using the position-type double vector obtained from the quadratic integration of the gravity vector. Figure 7 shows the convergence of the three attitudes during inertial frame coarse alignment based on 200 s of data before navigation. The pitch and roll converge quickly and stabilize within a few seconds, while the yaw oscillates for tens of seconds before leveling off. Compared to the analytical coarse alignment method under a static base, the inertial frame coarse alignment method is relatively more time-consuming.

We also included a 30 min coarse alignment and a 30 min fine alignment based on the GNSS position update classic Kalman filter (classic KF) to evaluate the coarse alignment accuracy. The initial parameters selected for the Kalman filter fine alignment are as follows:

P=diag(30″,30″,20′,0.1 m/s,0.1 m/s,0.1 m/s,1 m,1 m,1 m,0.005°/h,0.005°/h,0.005°/h,10 μg,10 μg,10 μg)^2  Q=diag(0.001°/h,0.001°/h,0.001°/h,10 μg/Hz,10 μg/Hz,10 μg/Hz)^2R=diag(10 m,10 m,30 m)^2

**Figure 8:**
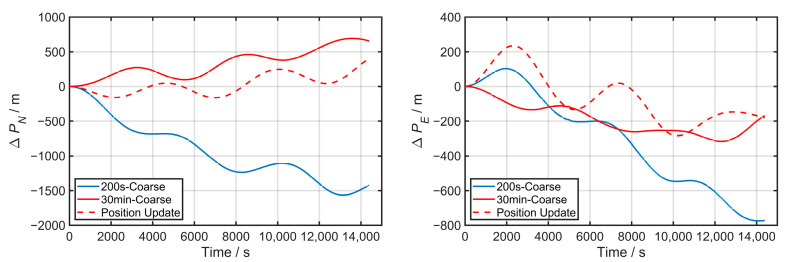
Three alignment strategies’ subsequent navigation error accumulation with time.

The subsequent navigation errors based on a 200 s coarse alignment, a 30 min coarse alignment, and the two-step method that includes a 200 s coarse alignment and a 30 min classic KF fine alignment are compared and presented in Figure 8.

The navigation error, Δ*p*, which indicates the pure inertial navigation position error, is adopted and calculated using Equation (20).

(20) $$\Delta p=\sqrt{{{\Delta p}_{N}}^{2}+{{\Delta p}_{E}}^{2}}$$

where ${\Delta p}_{N}$ and ${\Delta p}_{E}$ are the north and east navigation errors, respectively.

The maximum values for Δ*p* and the corresponding attitudes are listed in Table 1, where a minor navigation error indicates higher alignment accuracy, and obviously, the two-step method performs better.

As shown in Table 1, the attitude from the 200 s inertial frame coarse alignment is quite close to the two-step method in numerical terms, demonstrating the effective acquisition of the rough initial attitude of the vessel in a short time under mooring conditions, with the precision satisfying the small misalignment angle condition for further fine alignment.

As the coarse alignment time extends to 30 min, the difference in yaw is further reduced, and the maximum position error in 4 h of pure inertial navigation is significantly reduced from 1730 m to 737 m. This indicates that prolonging the coarse alignment time can improve the initial attitude precision to some extent. However, it is still inferior to the two-step method, which incorporates a Kalman filter fine alignment that reduces the position error to 434 m within the same time.

These results demonstrate that subsequent fine alignment remains essential for high-precision applications.

### 5.2. Test of Position Update Kalman Filter Fine Alignment under Swaying Base (I)

The experiments conducted in the previous section demonstrate the advantages of a two-step alignment method in terms of alignment accuracy within a similar limited time. In this section, the performance of the classic KF and the VCKF methods for fine alignment is further tested based on the approximate attitude achieved by inertial frame coarse alignment.

The data used in this section are the same as in Section 5.1, but the start time is adjusted to 1 h earlier. In this case, the first 1.5 h of data are utilized for initial alignment, while the following 4.0 h are used for navigation.

**Figure 9:**
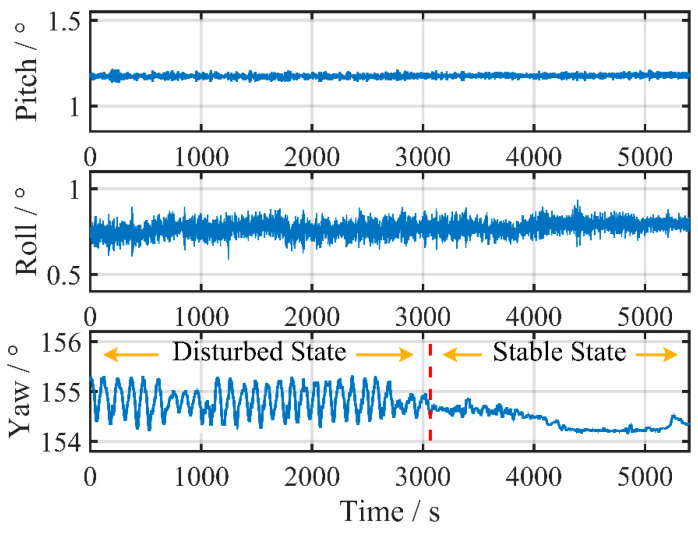
Vessel attitude during alignment in Test 1.

Figure 9 illustrates the change in carrier attitude during alignment. It can be observed that for the first 3000 s, there is periodic and continuous swaying interference in the azimuth. Subsequently, the interference disappears, and the observation environment remains relatively stable until entering the navigation state.

**Figure 10:**
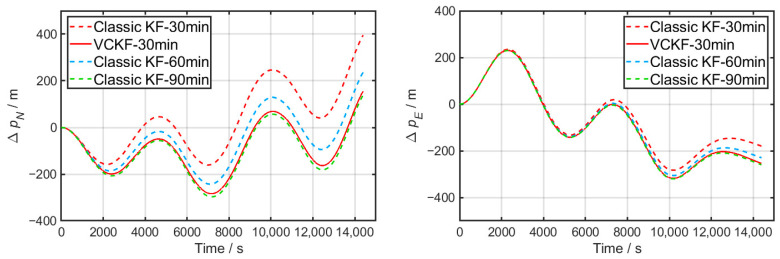
Accumulation of navigation errors corresponding to classic KF and VCKF for different time durations in Test 1.

During the test, the classic KF and VCKF methods employ the same parameters as in Section 5.1. Figure 10 depicts the position error resulting from 4 h of pure inertial navigation, comparing the results of different initial alignment durations and methods.

Figure 9 shows that interference in the azimuth occurs during the first half of the alignment. The 30 min fine alignment test only utilizes the second half of the data, resulting in a relatively stable environment for the entire fine alignment process despite the shorter duration. On the other hand, in the 60 min or 90 min tests, some or the entire first half of the data is included, leading to a period of disturbance before gradually transitioning into stability.

The final attitude after fine alignment is listed in Table 2, along with the maximum navigation position error calculated using Equation (20).

The results in Table 2 demonstrate that the classic KF remains a viable method for estimating misalignment despite interference. The maximum navigation error decreases as the fine alignment time is prolonged, eventually reducing to 325 m in 90 min. There is a trade-off between alignment speed and accuracy. Compared to a static base fine alignment, which typically takes around 10 min, the fine alignment on a mooring base takes significantly longer.

In contrast, the VCKF method achieves convergence to a maximum navigation error of 325 m in just 30 min, indicating a significant improvement in the convergence of azimuth misalignment. As the fine alignment time increases, the results remain stable, with only minor numerical fluctuations observed in both Euler angles and maximum navigation error.

This group of tests highlights the advantages of the newly suggested VCKF method in terms of rapidity, as the time required for fine alignment on a mooring base can be significantly reduced.

### 5.3. Test of Position Update Kalman Filter Fine Alignment under Swaying Base (II)

In the previous test, the navigation results from SINS appeared ideal for the insufficient orientation change during the mooring state. In this section, we conducted another test to evaluate the performance of different fine alignment methods in dissimilar interference environments and compared the pure navigation results in real sailing conditions.

The experiment in this section lasts approximately 6 h, with the vessel being in a mooring state for the first hour for initial alignment and then sailing for the next 5 h for SINS navigation.

**Figure 11:**
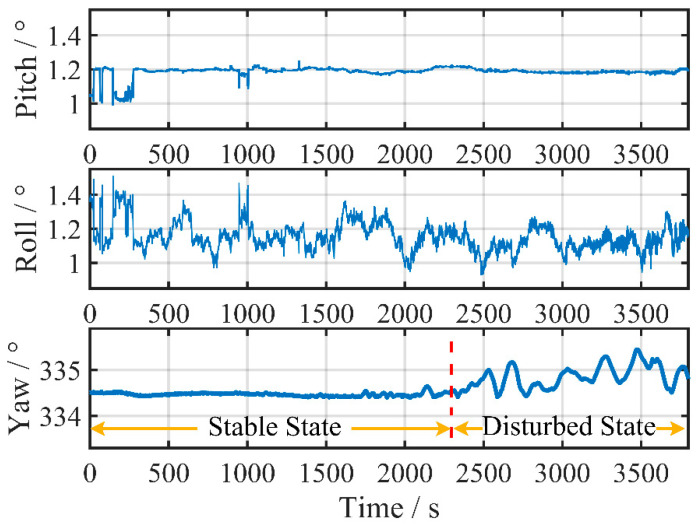
Vessel attitude during alignment in Test 2.

The vessel’s attitude in the mooring state is shown in Figure 11. Unlike the previous test, the interference occurs during the second half of the alignment process.

The identical two-step initial alignment strategy is adopted, where the initial fine alignment begins after the completion of the coarse alignment within the first 200 s. The filter parameters are set to the same as in Section 5.1.

**Figure 12:**
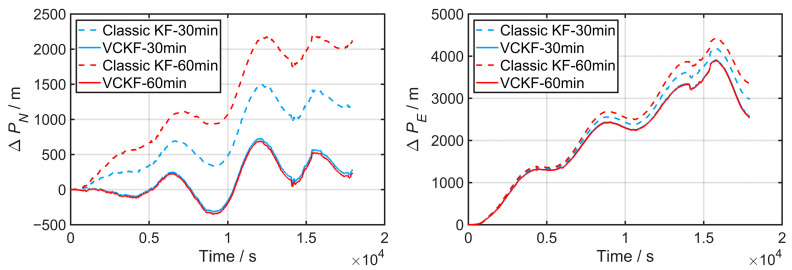
Accumulation of navigation errors corresponding to classic KF and VCKF for different time durations in Test 2.

The performance of the VCKF method and the classic KF method for fine alignment is compared in Figure 12 using pure inertial navigation error in the north and east directions, respectively. As illustrated, the error of SINS in the test has adequately aroused due to the heading changes in this stage, resulting in much more apparent navigation error accumulation compared to the previous test.

In addition, it can be observed in Figure 12 that the VCKF method achieved significantly smaller navigation error accumulation in both directions compared to the classic KF method.

The final attitude after fine alignment and the maximum navigation position error calculated by Equation (20) are listed in Table 3. Despite the presence of interference, the VCKF method consistently achieves stable results by the end of the fine alignment process, with subsequent maximum navigation errors consistently below 3950 m. In contrast, the maximum 5 h navigation error in the classic KF method increases from 4407 m to 4933 m when the fine alignment time extends from 30 to 60 min, contrary to our previous understanding.

**Figure 13:**
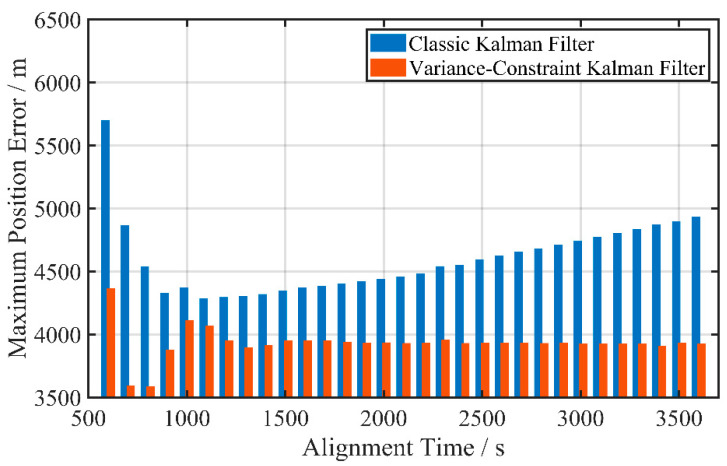
Variation in pure inertial navigation position error with alignment time for two fine alignment methods.

To exclude reason from individual data biases, some more tests are carried out in this section. By extending the fine alignment duration from 600 s to 3600 s in an equal interval of 100 s until the entire mooring stage is all involved in fine alignment, the change in the 5 h maximum navigation error for both the classic KF and VCKF methods within various alignment durations is shown in Figure 13.

Our previous expectation was that a longer alignment time would result in higher alignment quality. However, Figure 13 indicates a clear deviation between alignment time and navigation accuracy, suggesting that the results presented in Table 3 are not merely accidental. The interference that occurs in the second half has a non-negligible impact on misalignment estimation when the classic KF method is employed.

The result suggests a possible insufficiency of classic KF in the specific test scenarios of this section. Once the variances in states have decreased before the interference occurs, it is challenging for a classic KF to tackle the change with external observation updates, leading to performance degradation in error estimation. The longer the fine alignment lasts in a stable environment, the more adequately the misalignment angle variance converges, resulting in a larger attitude estimation deviation when the interference occurs.

In contrast, the suggested VCKF method for fine alignment demonstrates stronger robustness. The navigation error does not increase with the prolongation of fine alignment time in the presence of interference. In addition, the maximum navigation error in the VCKF fine alignment quickly converges in just 1500 s in the testing mooring state depicted in Figure 13, highlighting its advantage in terms of alignment speed.

## 6. Conclusions

This paper presents and evaluates a set of applicable alignment methods for the initial alignment of SINS in shipborne mooring states. The approaches start with a coarse alignment in the inertial frame to obtain an initial approximate attitude and then to estimate the misalignment error by the Kalman filter with external position updates. To enhance the estimation velocity and stability in fine alignment, we introduced a practical variance-constrained Kalman filter approach. This method sets a lower boundary based on a carefully designed relative convergence index, thereby improving the overall performance of the Kalman filter in the fine alignment phase.

The shipborne test results demonstrate that the inertial frame coarse alignment, based on a position-type double vector, can obtain a rough initial attitude within tens of seconds in moored states and on swaying bases, and the accuracy achieved meets the small misalignment angle condition requirements for subsequent fine alignment. The results of the fine alignment further indicate that, in a relatively stable environment, the classic Kalman filter and the newly proposed VCKF method yield similar final attitude and navigation accuracy. Still, notable differences are observed in terms of alignment speed. The VCKF method takes significantly less time to achieve the same alignment effect, demonstrating its advantage regarding alignment efficiency.

Furthermore, in terms of stability, the classic Kalman filter is susceptible to environmental disturbances, and interference that occurs during the end stage of alignment can significantly affect attitude determination. In contrast, the VCKF method exhibits more excellent stability even when interference occurs during the end stage of alignment, and no observable influence is found in the subsequent navigation results, highlighting the robustness of this method.

In practical applications, disturbances from the ocean and mooring environment are often unpredictable. The variance constraint method, as demonstrated in this study, proves to be an effective way to improve the performance of the Kalman filter in fine alignment when only external position observations are available, enabling rapid and stable attitude determination on sea mooring carriers.

## Figures and Tables

**Table 1:** Comparison of 200 s coarse, 30 min coarse, and 30 min position update alignment result.

Strategy	Pitch (°)	Roll (°)	Yaw (°)	4 h Maximum Position Error
200 s coarse	1.1736	0.7815	154.3175	1730.48
30 min coarse	1.1745	0.7832	154.3410	737.42
30 min position update	1.1740	0.7811	154.3385	434.42

**Table 2:** Alignment result of classic KF and VCKF for different time durations in Test 1.

Strategy	Pitch (°)	Roll (°)	Yaw (°)	4 h Maximum Position Error
30 min-Classic KF	1.1740	0.7811	154.3385	434.42
60 min-Classic KF	1.1740	0.7811	154.3367	331.76
90 min-Classic KF	1.1740	0.7811	154.3355	325.03
30 min-VCKF	1.1740	0.7811	154.3358	324.77
60 min-VCKF	1.1740	0.7811	154.3359	325.49
90 min-VCKF	1.1740	0.7811	154.3361	326.35

**Table 3:** Alignment result of classic KF and VCKF for different time durations in Test 2.

Strategy	Pitch (°)	Roll (°)	Yaw (°)	5 h Maximum Position Error
30 min Classic KF	1.1947	1.1965	334.7932	4406.98
60 min Classic KF	1.1945	1.1966	334.8023	4933.01
30 min VCKF	1.1949	1.1967	334.7831	3945.66
60 min VCKF	1.1950	1.1966	334.7826	3925.09

## Data Availability

No new data were created or analyzed in this study. Data sharing is not applicable to this article.
